# Comparison of the effect of post space preparation time on the apical seal of two different sealers

**DOI:** 10.1186/s12903-022-02367-z

**Published:** 2022-08-17

**Authors:** Neda Hajihassani, Navid Mohammadi, Ahmad Karimi Kelayeh, Shima Aalaei

**Affiliations:** 1 Department of Endodontics, Dental Caries Prevention Research Center, Qazvin University of Medical Sciences, Qazvin, Iran; 2 Children Growth Research Center, Research Institute for Prevention of Non-Communicable Diseases, Qazvin University of Medical Sciences, Qazvin, Iran; 3 Canada Optimax Access Consulting, Conquitlam, BC Canada; 4 Student Research Committee, Qazvin University of Medical Sciences, Qazvin, Iran; 5 Department of Prosthodontics, Dental Caries Prevention Research Center, Qazvin University of Medical Sciences, Bahonar Blvd., Qazvin, Iran

**Keywords:** AH plus sealer, Root canal obturation, Dental seal, Endoseal MTA sealer, Fluid filtration

## Abstract

**Background:**

The present study compared the effect of post space preparation time on the apical seal of two different sealers.

**Methods:**

In the in vitro study, 94 central incisors were used. After the samples’ root canal preparation, they were randomly assigned to four experimental groups (*n* = 21). The samples in groups 1 and 2 were obturated with AH Plus sealer, gutta-percha, and in groups 3 and 4 with Endoseal MTA bioceramic sealer and single cone technique. The post spaces in groups 1 and 3 were prepared immediately and in groups 2 and 4 with a delay. The samples were evaluated at 7-, 30-, and 90-day intervals for apical microleakage using the fluid filtration technique. The data were analyzed with SPSS 25, using three-way ANOVA and independent *t*-test.

**Results:**

The apical microleakage in groups 3 and 4, obturated with Endoseal MTA bioceramic sealer and prepared immediately and after a delay, respectively, was not significantly different between the interval times. In group 2, obturated with AH Plus sealer and prepared for post space with a delay, the apical microleakage was significantly less than all the other groups. Group 1, obturated with AH Plus sealer and prepared for post space immediately, exhibited the least microleakage after seven days, but its microleakage increased over time to reach the level of groups 3 and 4.

**Conclusion:**

According to the results, the apical microleakage in the AH + sealer group and the delayed post-space preparation method, was significantly less than all the other groups over time.

## Background

The goal of root canal obturation is the three-dimensional sealing of the root canal space to prevent penetration of microorganisms into the root canal(s), eliminate periapical lesions, or prevent their progression [1]. Most endodontic techniques for root canal obturation favor a principal material and a sealer. Sealers are necessary for all the obturation techniques and help achieve an impermeable seal [2]. Dentists routinely use the AH Plus resin sealer in different root canal obturation techniques due to its favorable characteristics, including adhesion to dentin and high sealing ability [3]. Therefore, it is a gold standard in comparative studies on newly introduced sealers [4].

The use of bioceramic sealers, especially Endoseal MTA sealer (MARUCHI Products, South Korea), has increased due to its favorable physical and chemical properties [5], including easy use, lack of shrinkage, good flowability, injection capacity, short setting time, minimal tooth discoloration, good adaptability, and the capacity to increase dentinal tubules’ biomineralization by MTA [6]. Most of the teeth treated endodontically need a post-and-core restoration [7]

It is important not to disrupt the integrity of the apical seal during post space preparation. There is controversy over how, when, and the amount of gutta-percha removed for post space preparation. Long et al*.* [8] and Reyhani et al*.* [9] reported no significant difference between the delayed and immediate post space preparation methods using bioceramic sealers. Nagas et al*.* [10] and Gungor et al*.* [11] reported low microleakage in the delayed post space preparation method with the use of AH Plus resin sealer; however, Kim et al*.* [12] reported that AH Plus sealer exhibited more microleakage, in delayed post space preparation method compared to immediate.

Padmanabhan et al*.* [13] and Dhaded et al*.* [14], too, reported a significantly better seal in the immediate method with both resin and bioceramic sealers. In most studies, the AH Plus sealer has exhibited superior sealing ability than the others.

There are discrepancies in the results of studies on microleakage of different bioceramic sealers compared to AH Plus sealer in immediate and delayed post space preparation methods. Besides, there is an ever-increasing use of bioceramic sealers in the single-cone obturation technique and immediate post space preparation method because it is time-saving. The differences between the results of different studies on microleakage can be attributed to differences in evaluation times. Since the setting degree of sealers depends on time, this study was undertaken to compare the microleakage of AH Plus (epoxy resin) and Endoseal MTA (bioceramic) sealers after immediate and delayed post space preparation at 7-, 30-, and 90-day intervals using the fluid filtration technique.

## Methods

In the present experimental study, 150 extracted human maxillary central incisors with a round cross-section were selected from the Department of Oral and Maxillofacial Surgery, School of Dentistry, Qazvin University of Medical Sciences. Considering the study by Gungor et al*.* [11] and at *α* = 0.05 and *β* = 0.2, the sample size was calculated at *n* = 94 using G*Power software (*n* = 21 in each study group plus a positive and a negative control tooth in each group). Teeth with fractures, caries, cracks, congenital anomalies, root canal curvature, open apex, and calcified root canal were excluded. The remaining teeth were evaluated under a dental operating microscope to ensure the absence of cracks. Calcification and the number of root canals were evaluated by radiography.

The remaining 94 teeth were immersed in 5.25% NaOCl for disinfection. The tooth crowns were removed blew the CEJ with a diamond disk (Jota, Germany) to leave 13 mm of root length from the cut surface to the apex. All the samples were prepared up to the WL with ProTaper Universal files (Dentsply, Maillefer, Ballaique, Switzerland) up to file F3 with a rotary motor (NSK, ENDO-MATE DT, Japan). Then they were obturated.

The samples were randomly assigned to four study groups (*n* = 21) and one positive and four negative control groups (*n* = 2). The samples in groups 1 and 2 were obturated with gutta-percha (DiaDent, Korea) and AH Plus (Dentsply, Detrey, Germany) sealer using lateral condensation method and with Endoseal MTA (Maruchi, Wonju, Korea) sealer in groups 3 and 4 using the matched single-cone technique.

In groups 1 and 3, the post spaces were prepared immediately with a #3 Peeso reamer (Mani, Japan) to leave 5 mm of gutta-percha in the apical area of the root canal. In groups 2 and 4, the samples were stored in physiologic serum (100% humidity) in an incubator at 37℃ for one week, and then the post spaces were prepared like groups 1 and 3.

In the positive control group, the root canals were debrided and shaped but were not obturated; the external surface of roots was not covered. The root canals were prepared in the negative control groups, and the external surface of the root, even the apical end, was covered with nail varnish. The root canals were prepared and obturated with gutta-percha (DiaDent, Korea) and AH Plus (Dentsply, Detrey, Germany) sealer or Endoseal MTA (Maruchi, Wonju, Korea) sealer using the matched single cone technique. Then the post space was prepared immediately or with a delay in each sealer group.

The coronal area of all the samples was covered with self-cured glass-ionomer (GC Fuji II, Japan) except for the positive control group. All the experimental samples’ external surfaces were covered with two layers of nail varnish except for 2 mm of the apical region.

The samples were then incubated at 98% relative humidity at 37ºC. Microleakage was evaluated at 7-, 30-, and 90-day intervals with the fluid filtration technique.

**Fig. 1:**
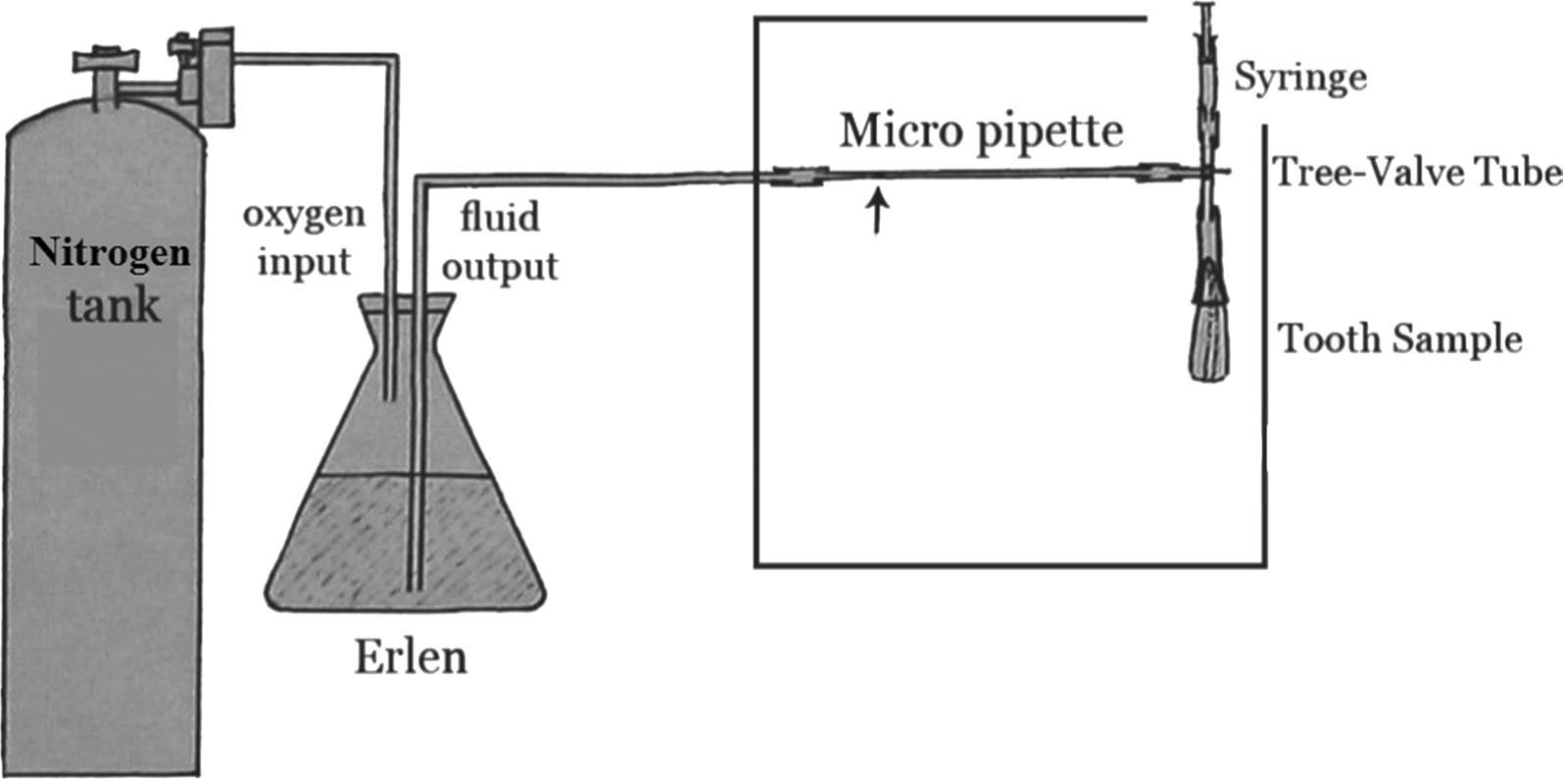
A Schematic Fluid Filteration System

In the fluid filteration technique method, the apical area of the samples is connected to a micropipette filled with a fluid, and the whole system is subjected to 0.5-bar nitrogen pressure [15]. The extent of water column displacement in the micropipette at the specified time interval indicates the extent of microleakage (Fig. 1). The system is accurate to μL level, and its unit is μL/minute/cm of water [15, 16].

### Data analysis

The data were coded and analyzed with SPSS 25. Descriptive and inferential statistics were used for data analysis. Table 1 presents the results in terms of means and standard deviations. The prerequisite of the statistical method (the normal distribution of the data) was assessed using the Kolmogorov–Smirnov test, and there was no significant difference between our data distribution and normal distribution.

**Table 1:** The means and standard deviations of microleakage in the study groups

Sealer	Post space preparation time	Microleakage evaluation time	Mean of microleakage (µL/min/cm H_2_O)	SD	*P*-value
AH Plus	Immediate (group 1)	7 days	0.24^a^	0.11	< 0.001
		30 days	0.45^c^	0.07	
		90 days	0.48^c^	0.11	
	Delayed (group 2)	7 days	0.36^b^	0.08	
		30 days	0.36^b^	0.08	
		90 days	0.36^b^	0.07	
Endoseal MTA	Immediate (group 3)	7 days	0.48^c^	0.08	
		30 days	0.50^c^	0.10	
		90 days	0.51^c^	0.11	
	Delayed (group 4)	7 days	0.51^c^	0.12	
		30 days	0.51^c^	0.07	
		90 days	0.52^c^	0.07	

Similar superscript letters indicate no statistically significant difference (*P* > 0.05)

Three-way ANOVA as a multivariable test was used to figure out the effect of three different qualitative variables (sealer type, time, and delay) on a quantitative response (microleakage).

We also used independent samples *t*-test to find out any significant difference between sealer types. Statistical significance was set at *P* < 0.05.

## Results

All the positive control group samples exhibited complete apical microleakage. None of the negative control group samples showed apical microleakage.

A three-way ANOVA showed that the sealer type, preparation time and delay had a significant association with microleakage (*P* < 0.001). All the interactions between the sealer type, preparation time, and the delay were associated with microleakage.

Table 1 shows the apical microleakage of sealers with the immediate and delayed post space preparation methods at 7-, 30-, and 90-day intervals.

To find the significant differences between sealer types, preparation times, and delays, we used the independent samples *t*-test. Based on the analysis, a comparison of the immediate and delayed post space preparation methods showed less microleakage with AH Plus cement in the immediate post space preparation method at a 7-day interval than in the delayed post space preparation method (*P* < 0.001). However, after 30 and 90 days, microleakage was significantly higher than in the delayed group (*P* < 0.001). In the delayed post-space preparation method, the apical microleakage was similar at the three-time intervals.

In the Endoseal MTA group, there were no significant differences in apical microleakage between the immediate and delayed post space preparation methods at the three-time intervals (*P* > 0.05).

There were no significant differences in apical microleakage of the two sealers in the immediate post space preparation between the 30- and 90-day intervals (*P* = 0.86, *P* = 0.39, respectively). However, at the 7-day interval, the AH Plus sealer exhibited less microleakage than the Endoseal MTA sealer (*P* < 0.001).

The difference in microleakage of the two sealers in the delayed post space preparation method at the three-time intervals was significantly different, and the AH Plus sealer exhibited less microleakage than Endoseal MTA at all the three-time intervals (*P* < 0.001).

Therefore, the apical microleakage of the AH Plus sealer with the immediate method was significantly lower than the other groups only at the 7-day interval, followed by the same sealer group with the delayed method, exhibiting no change at the three-time intervals. The other groups showed no significant differences.

## Discussion

The present study evaluated the microleakage of root canals obturated with AH Plus resin sealer and Endoseal MTA bioceramic sealer after immediate and delayed post space preparation procedures, with the fluid filtration technique. This technique has several advantages compared to dye penetration and microbial leakage methods. It is a quantitative technique, does not destroy the tooth, can be repeated over time [17], and is useful for longitudinal studies [18]. It does not need any specific indicator, with no relevant problems, including particle size and pH [18]. In addition, its results are very accurate [19], and very low volumes can be recorded with it [20].

In the AH plus sealer based on the time elapsed from the post space preparation, the difference between the immediate and delayed method is compared, which leads to a different result. In studies by Reyhani et al*.*, Kim et al*.*, Padmanabhan et al*.* and Dhaded et al*.*, [9, 12–14], the samples were evaluated at a 7-day interval, revealing less apical microleakage in the immediate method than in the delayed method. In studies by Nagas et al*.* and Abramovitz et al*.* [10, 21], the evaluation time was > 7 days. The microleakage of the delayed post space preparation time was less than that in the immediate method.

Concerning the ceramic sealer, the evaluation time of apical microleakage did not affect the difference in apical microleakage between the two post space preparation times, and the two methods showed similar microleakage at different time intervals, consistent with studies by Reyhani et al*.* and Padmanabhan et al*.* [9, 13].

The information mentioned above guides us toward a theory that can explain the discrepancies between the results of other studies and the microleakage of sealers. This theory runs as follows:

If the sealer polymerization is not complete when post space preparation is carried out, it flows and fills the apical gaps and spaces, decreasing microleakage. However, the stresses of post space preparation affect its polymerization and chemical properties, increasing microleakage over time. However, if the sealer polymerization is complete when the post space preparation is carried out, it does not flow, does not fill the apical gaps, and creates cracks; therefore, microleakage increases but does not change over time.

The above hypothesis explains the findings of the present study as follows.

The reason for differences in the apical microleakage of AH Plus sealer in the immediate post space preparation method at the three time intervals: However, stresses due to post space preparation affect the polymerization process and the chemical properties of the sealer [3], increasing microleakage in the long term.

The reason for no change in the apical microleakage of AH Plus sealer in the delayed post space preparation method at the three time intervals: Since the sealer has set completely [3] when the post space is prepared, it does not undergo significant chemical changes and polymerization process, resulting in no changes in microleakage over time.

The reason for differences in the apical microleakage of AH Plus sealer with the immediate and delayed methods at the 7-day interval: In the immediate post space preparation method, the sealer’s polymerization is not complete; therefore, it flows and fills the spaces [3, 22, 23], decreasing microleakage. However, in the delayed method, the sealer has already completed the polymerization process and cracks under the stresses of the post space preparation procedure, increasing microleakage [3, 14].

The reason for differences in the apical microleakage of AH Plus sealer with the immediate and delayed methods at the 30- and 90-day intervals: In the immediate post space preparation method, the sealer has not been polymerized completely, and stresses resulting from this procedure change the chemical properties of the sealer and affect the polymerization process, increasing microleakage over time [3] in contrast to the delayed method.

The reason for higher apical microleakage of Endoseal MTA sealer than AH Plus sealer in the immediate post space preparation method at the 7-day interval: Since the setting time of Endoseal MTA sealer is very short (< 4 min) [6], it is almost completely polymerized at the time of post space preparation. Therefore, it does not flow and does not fill the apical spaces and in contrast to the AH Plus sealer, becomes cracked.

The reason for no significant difference in the microleakage of Endoseal MTA sealer between the immediate and delayed post space preparation methods at the three time intervals: Since the setting time of this sealer is short, its setting reaction is complete in the delayed and immediate post space preparation methods, resulting in similar apical microleakage that does not change over time.

The reason for higher microleakage of Endoseal MTA sealer than AH Plus sealer in the delayed post space preparation method: In the delayed method, both sealers are almost completely polymerized. However, due to the generally better characteristics of the AH Plus sealer, it provides a better apical seal with lower microleakage. These characteristics include higher flowability, thixotropic behavior, lower film thickness, higher viscosity [24], chemical bond to dentine, self-etching properties [25], and better penetration into the dentinal tubules [26].

Dos Reis-Prado et al*.* [27] showed in a systematic review that microleakage in the delayed method was more than the immediate method because most studies included in this systematic review evaluated microleakage in less than seven days [3, 13, 14, 28–38]. However, in studies in which the evaluation time was > 7 days and < 30 days, the microleakage of the delayed and immediate method was similar [21, 22, 39–43]. In the two studies where the evaluation time was > 7 days [10, 21], microleakage in the immediate method was higher than that in the delayed method. Therefore, most studies included in the systematic review confirmed the hypothesis above. This hypothesis, too, should be further evaluated, similar to other hypotheses, so that it can be treated as a fact.

## Conclusion

According to the present study, the best time to prepare the post space is determined by the sealer’s setting time. The post space should be prepared after the complete setting of the sealer. Concerning sealers with a short setting time, such as Endoseal MTA, the post space preparation time does not affect the apical microleakage; however, in sealers with a long setting time, such as AH Plus, it is advisable to delay the post space preparation procedure until the sealer completes its setting reaction.

## Data Availability

All data generated or analysed during this study are included in this published article.
